# Sensitivity of human pleural mesothelioma to oncolytic measles virus depends on defects of the type I interferon response

**DOI:** 10.18632/oncotarget.6285

**Published:** 2015-11-02

**Authors:** Carole Achard, Nicolas Boisgerault, Tiphaine Delaunay, David Roulois, Steven Nedellec, Pierre-Joseph Royer, Mallory Pain, Chantal Combredet, Mariana Mesel-Lemoine, Laurent Cellerin, Antoine Magnan, Frédéric Tangy, Marc Grégoire, Jean-François Fonteneau

**Affiliations:** ^1^ INSERM, UMR892, Institut de Recherche en Santé de l'Université de Nantes, Nantes, France; ^2^ CNRS, UMR6299, Institut de Recherche en Santé de l'Université de Nantes, Nantes, France; ^3^ Université de Nantes, Nantes, France; ^4^ INSERM UMS016, SFR Santé, Nantes, France; ^5^ INSERM UMRS1087, Institut du Thorax, Nantes, France; ^6^ CNRS UMR3569, Unité de Génomique Virale et Vaccination, Institut Pasteur, Paris, France; ^7^ CHU de Nantes, Service d'Oncologie Médicale Thoracique et Digestive, Nantes, France; ^8^ CHU de Nantes, Service de Pneumologie, Nantes, France

**Keywords:** oncolytic virus, measles virus, oncolytic virotherapy, mesothelioma, type I interferon

## Abstract

Attenuated measles virus (MV) is currently being evaluated as an oncolytic virus in clinical trials and could represent a new therapeutic approach for malignant pleural mesothelioma (MPM). Herein, we screened the sensitivity to MV infection and replication of twenty-two human MPM cell lines and some healthy primary cells. We show that MV replicates in fifteen of the twenty-two MPM cell lines. Despite overexpression of CD46 by a majority of MPM cell lines compared to healthy cells, we found that the sensitivity to MV replication did not correlate with this overexpression. We then evaluated the antiviral type I interferon (IFN) responses of MPM cell lines and healthy cells. We found that healthy cells and the seven insensitive MPM cell lines developed a type I IFN response in presence of the virus, thereby inhibiting replication. In contrast, eleven of the fifteen sensitive MPM cell lines were unable to develop a complete type I IFN response in presence of MV. Finally, we show that addition of type I IFN onto MV sensitive tumor cell lines inhibits replication. These results demonstrate that defects in type I IFN response are frequent in MPM and that MV takes advantage of these defects to exert oncolytic activity.

## INTRODUCTION

Antitumor virotherapy using oncolytic viruses is a developing strategy to treat cancer [1]. Among oncolytic viruses, attenuated strains of measles virus (MV) have been shown to infect and kill a large variety of tumor cell lines [2, 3]. Phase I clinical trials using the Edmonston strain of MV have shown clinical benefits for the treatment of cutaneous T cell lymphoma [4], ovarian cancer [5, 6] and disseminated multiple myeloma [1]. The Edmonston MV is also currently being evaluated in on-going phase I clinical trials for the treatment of squamous cell carcinoma of the head and the neck, glioma and mesothelioma by the group of Stephen J. Russell at the Mayo Clinic [1].

Schwarz and Edmonston attenuated strains of MV use the CD46 molecule as the major receptor to infect human cells, unlike the pathogenic strains that mainly use the CD150 molecule [7–9]. The membrane cofactor protein CD46 is ubiquitously expressed at a low level by all nucleated cells and blocks the complement cascade at the C3 activation stage [10]. CD46 is often overexpressed on tumor cells of many cancer types to escape complement-mediated cytotoxicity [11, 12]. This expression at high density confers to attenuated MV a natural tropism for tumor cells. In fact, above a certain threshold of CD46 expression, the killing and syncitia formation mediated by MV infection increase dramatically [7]. Healthy cells that express a low level of CD46 are not infected [13]. Recently, nectin-4 has been described as a receptor for attenuated and wild-type MV, but its implication in the oncolytic activity of MV is still to be determined [14, 15].

The overexpression of CD46 is probably not the only factor that conditions the ability of MV to preferentially replicate in and kill tumor cells. In fact, there is now evidence that host-cell translational activity upon viral replication [16] and defects in the capacity of tumor cells to develop an antiviral innate immune response [17, 18] participate in MV oncolytic activity. All nucleated cells are equipped with intracytoplasmic sensors that are able to detect viral infection [19]. In the case of paramyxoviruses, helicases such as RIG-I and MDA5 detect viral RNA and induce the secretion of type I IFN, mainly IFN-β in non-immune cells that protect infected and neighboring cells from viral replication. Indeed, exposure to type I IFN induces in cells expressing the IFN-α/β receptors IFNAR1/IFNAR2 the expression of hundreds of IFN-sensitive genes (ISG) that exert anti-viral activity [19]. Among these, the IFN-induced GTP-binding protein Mx1 is able to inhibit the early steps of viral replication by interfering with the formation of the ribonucleoproteic complex [20].

We have previously shown that the Schwarz attenuated strain of MV induces immunogenic cell death of malignant pleural mesothelioma (MPM) cells [21, 22]. In this study, we screened the sensitivity to MV infection and replication of twenty-two MPM cell lines established in our laboratory [23], and four different types of primary healthy cells. We found that fifteen MPM cell lines were sensitive to MV replication. We then measured the cell surface expression of CD46, nectin-4 and CD150, the three known MV receptors. We found that CD46 was often overexpressed by MPM cell lines compared with healthy primary cells and was used as an entry receptor. However, we failed to observe a correlation between the level of CD46 expression and the sensitivity of MPM tumor cell lines to MV replication. We then analyzed the capacity of the different MPM cell lines to develop a type I and type III IFN response after exposure to MV. We found that their sensitivity to MV replication was strongly related to their type I IFN response.

## RESULTS

### Sensitivity of MPM and healthy primary cells to MV infection

**Figure 1:**
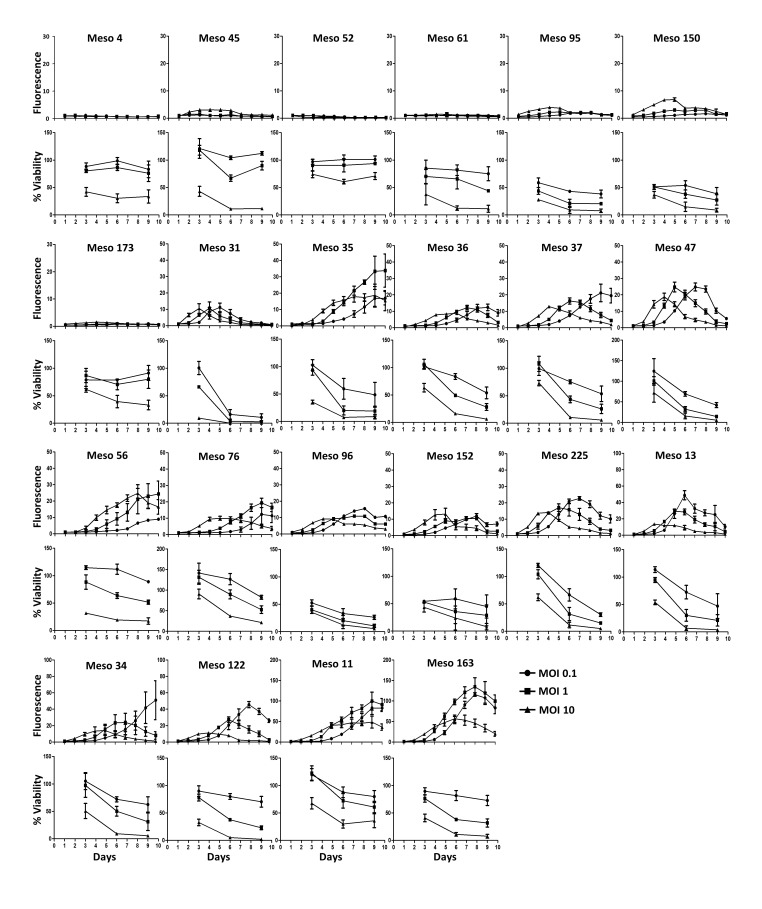
A majority of MPM tumor cell lines are sensitive to MV replication and oncolytic activity MPM cell lines were infected with MV-ch or MV at different MOI: 0.1 (circles), 1 (squares) and 10 (triangles). MV-ch and MV were used for the MV replication assay and the cell viability assay, respectively. The fluorescence values correspond to the ratio between the fluorescence measured in infected tumor cells and non-infected cells. Cell viability is expressed as a percentage compared to non-infected cells. Results are expressed as the mean ±SEM of three independent experiments.

To determine the sensitivity of a large number of MPM cell lines to MV infection and replication, we set up an assay using a recombinant MV encoding the cherry fluorescent protein (MV-ch). By measuring fluorescence at 610 nm, we followed daily MV-ch replication in twenty-two MPM cell lines exposed to different multiplicities of viral infection (MOI) (Figure 1). Simultaneously, we quantified cell viability using the UptiBlue^TM^ assay that is based on their metabolic activity. We also filmed by time-lapse microscopy some of the MPM cell lines exposed to MV encoding the enhanced green fluorescent protein (MV-eGFP) at an MOI = 1 (Videos 1–9). We observed no or low replication of MV in seven tumor cell lines. For five of these, no MV replication was observed: Meso4 (Video 1), Meso45, Meso52 (Video 2), Meso61 and Meso173, but their viability decreased at the highest MOI. For the other two tumor cell lines, Meso95 and Meso150 (Video 3), MV replicated in a few cells, which then induced apoptosis of the neighboring non-infected cells.

In the fifteen other MPM cell lines we observed a strong replication of MV that led to cell death, with kinetics depending on the cell lines. MPM cell lines such as Meso31, Meso35, Meso152 and Meso225 underwent cell death quite fast after infection (Videos 4, 5, 6, 7), whereas for other sensitive cell lines, such as Meso11 and Meso163, cell death was slower (Videos 8, 9). This delay allows these latter cell lines to accumulate fluorescence resulting from viral replication. We also observed that infection was usually accompanied by the formation of syncytia (Videos 4, 6, 7, 8), but not in all tumor cell lines (Videos 5, 9). Green fluorescence measured in the videos (Supplemental figure 1) was very similar to the results obtained with cherry fluorescence measured using MV-ch (Figure 1). We observed no replication or very limited replication in Meso4, 52 and 150, a replication that stopped around day 3 and day 4 for Meso31, 35, 152 and 225, and a replication that continued after day 5 for Meso11 and 163. Altogether, these results show that approximately 70% of MPM tumor cell lines are sensitive to the replication and oncolytic activity of Schwarz MV.

**Figure 2:**
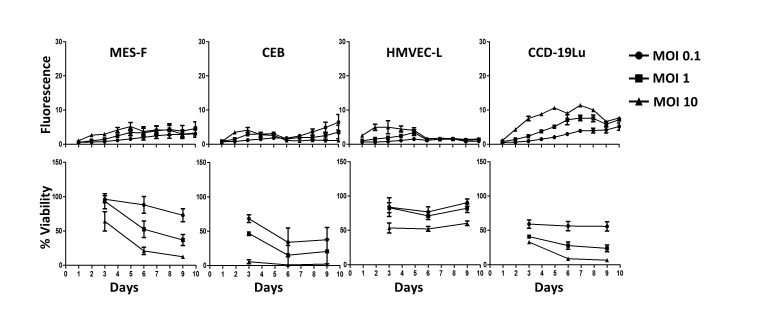
Healthy cells are not sensitive to MV replication 4 types of healthy cells (mesothelial cells MES-F, bronchial epithelial cells CEB, pulmonary endothelial cells HMVEC-L and pulmonary fibroblasts CCD-19Lu) were infected with MV-ch or MV at different MOI: 0.1 (circles), 1 (squares) and 10 (triangles). MV-ch and MV were used for the MV replication assay and the cell viability assay, respectively. The fluorescence values correspond to the ratio between the fluorescence measured in infected tumor cells and non-infected cells. The cell viability is expressed as a percentage compared to non-infected cells. Results are expressed as the mean ±SEM of three independent experiments.

Using the same techniques, we also measured the sensitivity to MV infection of four different human primary healthy cell types: peritoneal mesothelial cells (MES-F), bronchial epithelial cells (CEB), pulmonary endothelial cells (HMVEC-L) and lung fibroblasts (CCD-19Lu) (Figure 2). We observed no infection of CEB (Supplemental video 10) and a very limited infection with no syncytia formation for MES-F (Supplemental video 11), HMVEC-L, and CCD-19Lu cells. The viability of these primary cells decreased, especially at the highest MOI.

### Expression of CD46, CD150 and nectin-4 by MPM and healthy primary cells

**Figure 3:**
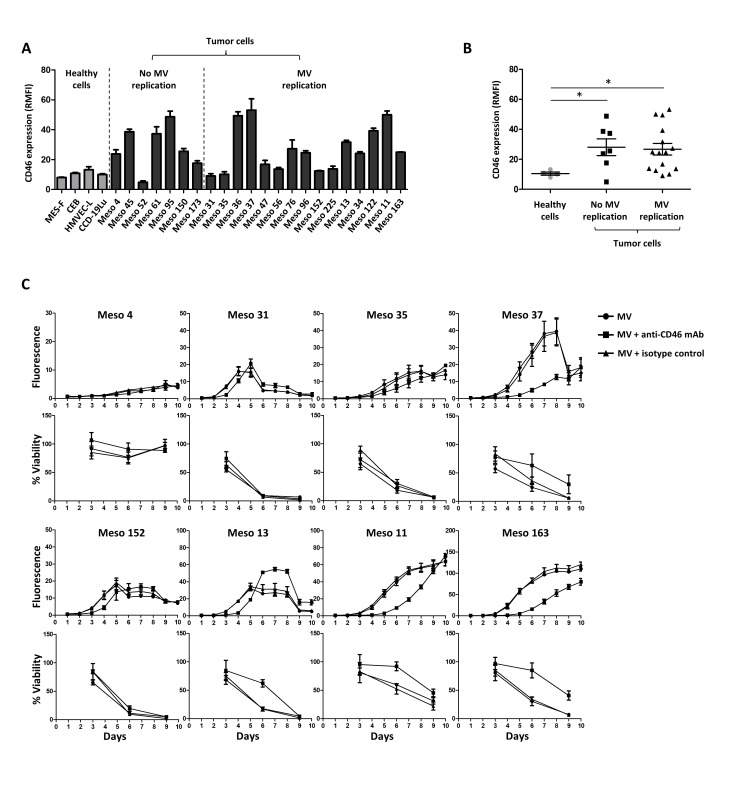
Absence of correlation between CD46 surface expression and MPM sensitivity to MV replication (**A**) Expression of CD46 is measured on the cell surface by flow cytometry. Results are expressed as RMFI ± SEM of three independent experiments. (**B**) Scatter plot representation of the CD46 expression on healthy and tumor cells surfaces. Each point represents the mean of RMFI obtained in three independent experiments. * *p* < 0.05, Mann-Whitney test. (**C**) MV replication and cell viability were assessed after MV-ch or MV infection, respectively (MOI = 1) in the presence or absence of an anti-CD46 blocking mAb. An isotype was used as a control. The fluorescence values correspond to the ratio between the fluorescence measured in infected tumor cells and non-infected cells. The cell viability is expressed as a percentage compared to non-infected cells. Results are expressed as the mean ±SEM of three independent experiments.

We measured the expression of known MV receptors (CD46, CD150 and nectin-4) on the surface of MPM and healthy primary cells. The majority of MPM cells expressed higher levels of CD46 than healthy primary cells (Figure 3A–3B). However, no statistical difference was observed between MPM cells sensitive or not to MV infection (Figure 3B). We did not detect any expression of CD150 or nectin-4 (Supplemental figure 2), which are receptors for the pathogenic as well as vaccine MV strains [14, 15, 24]. Altogether these data show that a majority of tumor MPM cell lines overexpress CD46 as the only known MV receptor and that sensitivity of these cells to MV infection is not related to CD46 expression.

### MV uses CD46 to infect MPM tumor cells

To determine whether CD46 plays a role in MPM cell infection, we exposed eight MPM cell lines to MV-ch in the presence of anti-CD46 mAb or isotype control mAb (Figure 3C). On Meso4, which is not sensitive to MV infection, the anti-CD46 mAb had no effect on replication, but slightly increased cell viability. On the seven other MV-sensitive cell lines, the anti-CD46 mAb significantly delayed MV replication and cell death. These delays were similar to the shifts observed between infection at MOI = 1 and 0.1 (Figure 1A), suggesting that the anti-CD46 mAb inhibited approximately 90% of the infection. This demonstrates that CD46 is required for MV infection of MPM tumor cells.

### IFN type I response prevents MV replication in MPM tumor cells and healthy primary cells

**Figure 4:**
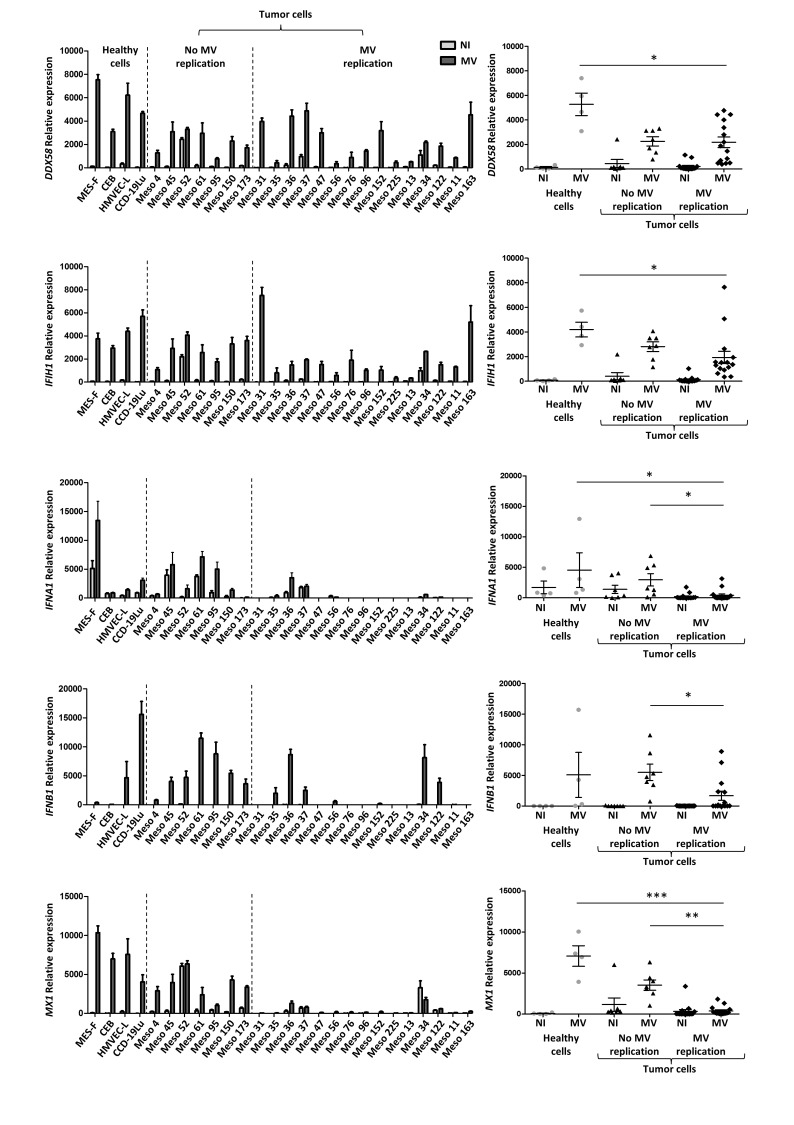
The sensitivity to MV infection depends on defects of the antiviral type I IFN response The expression of five genes implicated in the antiviral type I IFN response was analyzed by RT-qPCR 72 hours after MV infection of tumor and healthy cells (MOI = 1). The expression is expressed as relative expression compared to *RPLPO* gene expression. Non-infected cells (NI) are in light gray and infected cells (MV) are in dark gray. The *DDX58* and *IFIH1* genes code for RIG-I and MDA5 proteins, respectively. The *IFNA1* gene codes for IFN-α, *IFNB1* for IFN-β, and *Mx1* for Mx1 protein. For each gene, a histogram shows the expression by each cell line, and a scatter plot shows the expression by groups (healthy cells, tumor cells with no MV replication, tumor cells with MV replication). Results are expressed as the mean ±SEM of three independent experiments. * *p* < 0.05; **p < 0.01; ***p < 0.001, one-way ANOVA (Kruskal-Wallis).

Since the sensitivity of MPM tumor cell lines to MV replication did not correlate with the CD46 expression level (Figure 3B), we sought to identify other factors that condition sensitivity to MV replication. We investigated the activation of antiviral type I and III IFN response by tumor cell lines and healthy primary cells exposed for 72 hours to MV by analyzing the expression of five specific genes by RT-qPCR (Figure 4).

We first analyzed the expression of two helicase genes: the *DDX58* gene that encodes the retinoic acid-inducible gene-1 protein (RIG-I) and the *IFIH1* gene that encodes melanoma differentiation-associated protein 5 (MDA5). These two proteins are intracytoplasmic sensors of viral ssRNA and dsRNA, able to induce type I IFN response against MV [25]. We observed that following MV exposure, the expression of both genes was increased in all tumor cell lines and healthy primary cells, thus indicating that MV was detected by all these cells (Figure 4).

We then looked at the expression of two type I IFN genes: *IFNA1* and *IFNB1* that encode IFN-α and IFN-β, respectively (Figure 4). Constitutive expression of *IFNA1* was observed in the absence of MV in all healthy cells and in all insensitive tumor cell lines, with the exception of Meso173. *IFNA1* expression was increased in all these cell lines in the presence of the virus, with the exception of CEB. In the fifteen sensitive tumor cell lines, a weak constitutive expression of *IFNA1* in the absence of the virus was found in six tumor cell lines (Meso35, 36, 37, 56, 34 and 122) and was increased in the presence of MV in four of these (Meso35, 36, 34 and 122). In the nine other sensitive tumor cell lines, we never detected *IFNA1* expression, either in the presence or absence of MV. Regarding *IFNB1*, a weak constitutive expression in the absence of the virus was detected only in the insensitive Meso52 cell line (Figure 4). In the presence of MV, we measured a significant induction of *IFNB1* expression in all insensitive tumor cells lines and in all healthy cells, even in CEB in which *IFNB1* expression was increased 20-fold. In contrast, seven out of the fifteen sensitive tumor cell lines expressed *IFNB1* in response to the virus (Meso35, 36, 37, 56, 152, 34 and 122), whereas the eight other sensitive tumor cell lines did not. We also measured the expression of the *IFNL1* gene that encodes the type III IFN, IFN-λ1 (Supplemental Figure 3). In contrast to type I IFN, all tumor cell lines were able to express this gene in the presence of the virus with no significant differences whether MV-sensitive or not.

Finally, we measured the expression of the *MX1* gene that encodes the interferon-induced GTP-binding protein Mx1. The *MX1* gene is an ISG that is expressed following signaling from the type I IFN receptor, IFNAR1/IFNAR2. Among healthy primary cells, we found a weak constitutive expression of *MX1* only in HMVEC-L (Figure 4E). In the presence of MV, a strong increase of *MX1* expression was induced in all healthy cells. Similarly, in MV-insensitive MPM cell lines, the weak constitutive expression of *MX1* was highly increased after MV addition. Conversely, among the fifteen MV-sensitive MPM cell lines, we found no constitutive expression of *MX1* in eleven, a weak constitutive expression in three (Meso36, 37, 122) and a strong constitutive expression only for one (Meso34). In the presence of MV, we observed a significant increase of *MX1* expression only for Meso36. The *MX1* expression did not change for all the other sensitive cell lines.

These results indicate that cells that are able to develop a complete type I IFN response, whether they are healthy primary or tumor cells, are not sensitive to MV infection. On the contrary, tumor cell lines that are unable to develop a type I IFN response are sensitive to MV infection, with the four exceptions, Meso36, 37, 34 and 122, which express *IFNA1*, *IFNB1* and *MX1* and are sensitive to MV replication. These results also signify that the capacity to achieve a complete type I IFN response is defective in numerous MPM cell lines.

**Figure 5:**
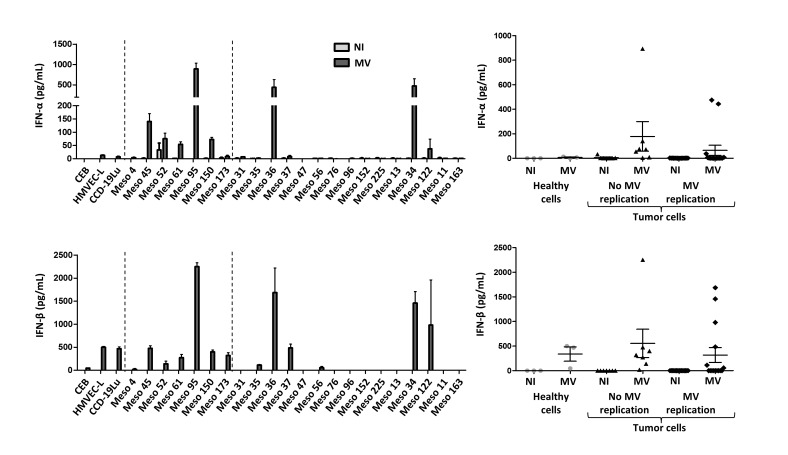
Secretion of the type I IFN, IFN-α and IFN-β, after MV infection IFN-α and IFN-β secretion was measured by ELISA, 72 hours after MV infection of tumor and healthy cells (MOI = 1). Non-infected cells (NI) are in light gray and infected cells (MV) are in dark gray. For each IFN, a histogram shows the expression by each cell line, and a scatter plot shows the expression by groups (healthy cells, tumor cells with no MV replication, tumor cells with MV replication). Results are expressed as the mean ±SEM of three independent experiments.

We then sought to confirm these results by measuring IFN-α and IFN-β secretion by ELISA in the culture supernatants (Figure 5). Regarding IFN-α,we did not detect significant secretion in the supernatants of tumor cell lines and healthy primary cell cultures in the absence of MV, except for Meso52 (Figure 5). This suggests that the IFN-α mRNA observed by RT-qPCR in several tumor cell lines in the absence of the virus was either not translated into proteins or resulted in levels undetectable by ELISA. In the presence of MV, IFN-α was significantly secreted by all insensitive cell lines and by two out of three healthy primary cell cultures, whereas only four out of the fifteen sensitive tumor cell lines (Meso36, 37, 34 and 122) secreted IFN-α. Regarding IFN-β, the results obtained by RT-qPCR were confirmed with the observation that this cytokine was secreted by all insensitive cell lines and healthy primary cells cultures, and by only six out of the fifteen sensitive cell lines (Meso35, 36, 37, 56, 34 and 122) (Figure 5). Interestingly, the four sensitive cell lines secreting IFN-α in the presence of the virus also secreted IFN-β.

**Figure 6:**
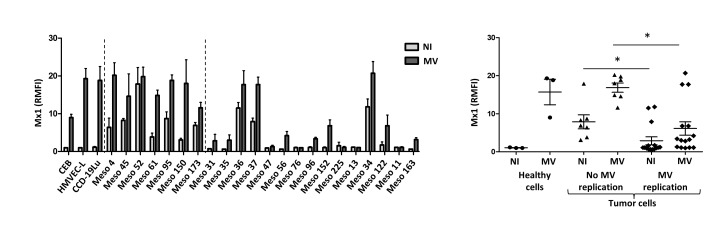
Sensitivity to MV replication correlates with the absence of expression of the antiviral protein Mx1 Intracytoplasmic Mx1 staining was performed on non-infected (NI) or on MV-infected tumor cells (MOI = 1, 72h) and fluorescence was analyzed by flow cytometry. The histogram represents the expression of Mx1 (RMFI) by each cell line (NI is in light gray and MV in dark gray), and the scatter plot shows Mx1 expression by groups (healthy cells, tumor cells with no MV replication, tumor cells with MV replication). Results are expressed as the mean ±SEM of three independent experiments. *p < 0.05, One-way ANOVA (Kruskal-Wallis).

We also measured the cytoplasmic expression of Mx1 protein by flow cytometry in tumor cell lines and healthy primary cells (Figure 6). We found high levels of Mx1 in the cytoplasm of healthy primary cells only when they were exposed to MV. In all insensitive tumor cell lines, we found cytoplasmic Mx1 in non-infected cells that was increased in the presence of MV, except for Meso52 where Mx1 was already present in equally high amounts in absence of the virus. For the fifteen tumor cell lines sensitive to MV replication, cytoplasmic Mx1 was not detected in the absence of MV except for four cell lines (Meso34, 36, 37 and 122). In the presence of MV, six out of the fifteen sensitive cell lines did not express Mx1 in their cytoplasm (Meso31, 47, 76, 225, 13 and 11), five expressed low levels in a fraction of the cells (Meso35, 56, 96, 152, and 163) and the last three expressed high levels of Mx1 in 100% of the cells (Meso34, 36, 37 and 122) similarly to what was observed in insensitive tumor cell lines.

Overall, these results show that tumor cells that constitutively express Mx1 are not sensitive to MV replication, with the exception of Meso34, 36, 37 and 122. They also demonstrate that the cells that fail to develop a complete type I IFN response, illustrated by the absence of IFN-α, IFN-β and Mx1 expression, are sensitive to MV replication with the same exceptions: Meso34, 36, 37 and 122.

### IFN-α and IFN-β inhibit MV replication in MPM cell lines sensitive to MV replication

**Figure 7:**
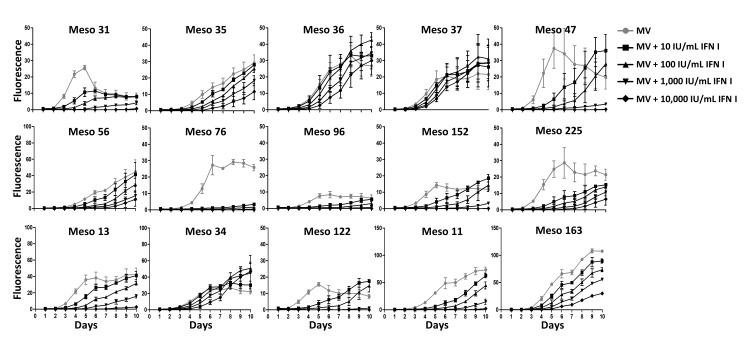
Type I IFN inhibit MV replication in the majority of MV-sensitive tumor cell lines MPM cell lines were infected with MV-ch (MOI = 1) in the presence of different amounts of IFN I (IFN-α and IFN-β) for MV replication assay. The fluorescence values correspond to the ratio between the fluorescence measured in infected tumor cells in the presence or absence of type I IFN and the non-infected cells. Data represent the mean ±SEM of three independent experiments.

In the last set of experiments we tested the effect of type I IFN (IFN-α and IFN-β) on MV replication in sensitive tumor cell lines. We first measured the expression of IFNAR1 and IFNAR2 in all MPM cells lines. A low expression was found on the surface of all tumor cell lines (data not shown). We then exposed the fifteen MPM cell lines sensitive to MV to increasing amounts of IFN-α and IFN-β and measured MV replication using MV-ch (Figure 7). MV replication was reduced in a dose-dependent manner in all tumor cell lines. Interestingly, the inhibition was lower in three out of the four MPM cell lines that express IFN-α, IFN-β and Mx1 constitutively (Meso34, 36 and 37), whereas the inhibition was more profound in tumor cell lines that did not develop a type I IFN response in the presence of the virus, such as Meso31, 47, 76, 225 and 11. In addition, exposure to IFN-α and IFN-β induced a strong expression of Mx1 in all of these fifteen sensitive tumor cell lines (data not shown). These results show that the majority of MPM cell lines that are sensitive to MV replication would not be able to replicate the virus if they were able to produce their own type I IFN in response to the virus.

## DISCUSSION

In this study, we measured the sensitivity of a large panel of human MPM tumor cell lines and of different types of healthy cells to the oncolytic activity of the Schwarz vaccine strain of MV. We found that fifteen out of twenty-two MPM tumor cell lines are sensitive to MV replication and cytopathic effect, whereas replication was limited or absent in the four types of healthy cells (fibroblasts, mesothelial, bronchial and endothelial cells) as well as in the seven remaining MPM tumor cell lines. Our data also show that the sensitivity of tumor cell lines is not correlated to overexpression of the MV cell receptor CD46, contrary to what has been described in the literature [3, 7]. Nevertheless, the virus needs CD46 to enter the cells. In a search for factors that affect the sensitivity of MPM tumor cells to MV oncolytic activity, we studied the innate antiviral type I and III IFN immune response of these cells. Our results suggest that MV enters into all tumor cell lines and healthy cells, since we observed the upregulation of genes encoding the cytoplasmic viral sensors RIG-I and MDA5 in the presence of the virus. However, we observed type I IFN production (IFN-α and -β) mainly by insensitive tumor cell lines and healthy cells exposed to MV. More strikingly, when we analyzed the expression of the ISG Mx1, we found this protein in the cytoplasm of all insensitive tumor cell lines and healthy cells exposed to MV. In contrast, eleven out of fifteen sensitive tumor cell lines were unable to express a high level of Mx1 in response to MV. Overall, this study suggests that about 70% of MPM patients are potentially sensitive to MV oncolytic activity and that this activity depends on defects in the intracellular innate antiviral response in MPM tumor cells rather than on CD46 overexpression on the cell surface.

We observed that exposure of insensitive tumor cell lines or healthy cells to high titers of MV (MOI = 10) results in viability loss for the majority of them. This is likely due to the induction of the innate antiviral immune response that is known to affect viability, notably by limiting host translational activity or by inducing apoptosis [19]. This is particularly true for the MPM tumor cell lines Meso95 and Meso150. For these cells, we observed that infection of a limited number of cells induces apoptosis of neighboring non-infected cells (Supplemental video 2). Apoptosis of non-infected cells was also observed at the lowest MOI, suggesting that a few infected cells are sufficient to induce apoptosis of neighboring cells. We classified Meso95 and Meso150 as tumor cell lines that are insensitive to MV replication. However, they could be considered as sensitive to the oncolytic activity of MV, even if MV replication is limited to a few cells. Overall, these results show that seventeen out of the twenty-two studied MPM tumor cell lines (77%) are sensitive to the oncolytic activity of MV. Furthermore, MV still has an effect on insensitive tumor cell lines by inducing a strong type I IFN response that could be beneficial for the patient by increasing tumor immunogenicity [26–28].

Two other groups recently studied the implication of the intracellular antiviral response on the sensitivity of tumor cell lines to MV oncolytic activity [16–18]. Berchtold and collaborators studied the sensitivity of eight human sarcoma tumor cell lines to the Schwarz strain of MV [18]. They found that five out of eight tumor cell lines were sensitive, whereas the remaining three were not. They then observed that the three insensitive cell lines express on their surface a lower level of CD46 molecules compared to the five sensitive cell lines, a result compatible with the accepted view that the oncolytic activity of MV depends on overexpression of CD46 by tumor cells [3, 7]. They also analyzed type I IFN response in tumor cells exposed to MV. When they analyzed the completion of this response by measuring expression of the ISG *IFIT1*, they found that it was expressed in the three insensitive cell lines and only in one out of the five sensitive cell lines. This last result matches our results that were obtained on a larger series of tumor cell lines and also on some healthy primary cells. The same group confirmed this result in an additional study where they analyzed type I IFN response in five MV-insensitive tumor cell lines compared to one sensitive tumor cell line [17]. They found that four out of the five insensitive tumor cell lines expressed IFIT1 in the presence of the virus, whereas the remaining insensitive cell line and the sensitive one did not develop a type I IFN response. The MV receptor level was not measured in this study. Finally, Patel and collaborators studied the sensitivity of seven human lung adenocarcinoma cell lines and two types of healthy cells to the Edmonston strain of MV [16]. They also analyzed PKR antiviral activity, whose expression is dependent on the type I IFN response, in two sensitive and one insensitive tumor cell line. Their results suggest that the PKR antiviral activity plays a role in the limited infection of the resistant tumor cell line by limiting the host transcriptional activity. Overall, our study performed on twenty-two MPM cell lines and four types of primary cells confirms and extends the previous conclusions obtained on a limited number of tumor cell lines that the type I IFN response in tumor cells affects their sensitivity to MV oncolytic activity. Furthermore, we clearly show that type I IFN defects play a greater role than CD46 overexpression.

We observed that some tumor cell lines that appear to develop a type I IFN response are nevertheless sensitive to MV oncolytic activity, as also observed in other studies [16–18]. This observation could be explained by the presence of two cellular subpopulations in these tumor cell lines: one sensitive subpopulation unable to express ISG in the presence of the virus, and one insensitive subpopulation competent in type I IFN response. However, we ruled out this hypothesis since we observed only one Mx1-positive population in these tumor cell lines in the presence of MV. This suggests that in sensitive tumor cell lines that produce type I IFN and express Mx1 in response to MV, other ISG involved in the inhibition of MV replication are missing. Indeed, while hundreds of ISG have been identified, we only studied Mx1 whose role in MV replication is not well characterized, especially with attenuated strains [29, 30]. Furthermore, this activity is likely to be cell-type dependent. It is thus possible that Mx1 antiviral activity acts in combination with other ISG that are not expressed in the four sensitive tumor cell lines that produce type I IFN and express Mx1 in response to MV, whereas these required ISG are expressed in the seven insensitive tumor cell lines, thereby blocking MV replication. In support of this hypothesis, it was demonstrated in a study that analyzed expression of 380 ISG in response to a panel of viruses that most ISG do not inhibit viral replication when expressed individually, and that antiviral activity is observed when ISG are expressed in combination [31, 32].

We thought to confirm these results *in vivo* with experiments on human MPM tumors engrafted in immunodeficient mice as we previously did for colon and lung adenocarcinoma [33]. However, these models are very different from what may happen in immunocompetent patients. Indeed, in these models, healthy mouse cells do not express receptors for MV and would not produce type I IFN. In addition, there is no immune system in these mice to respond to type I IFN produced by insensitive tumor cells. Thus, an immunocompetent animal model for mesothelioma needs to be developed to extend this study *in vivo*.

MV is currently being evaluated in clinical trials for the treatment of different types of cancers [1] and the first published results are promising [4–6, 34]. In our study, we define more precisely the mechanisms that dictate the sensitivity of MPM tumor cells to MV. These mechanisms should be taken into consideration to analyze the clinical outcome of MV-based virotherapy. It would be probably informative to know the type I IFN response capability of tumor cells from responding patients to better understand the efficacy of this therapeutic strategy.

## MATERIALS AND METHODS

### Cell culture

Human MPM cell lines (from Meso4 to Meso225) were established in our laboratory from pleural effusions collected by thoracocentesis, and genetically characterized [23]. All patients gave their informed consent. All cell lines were maintained in RPMI-1640 medium supplemented with 10% heat-inactivated fetal calf serum, 100U/mL penicillin, 100μg/mL streptomycin and 2mM L-glutamine (all reagents from Gibco-Invitrogen) and cultured at 37°C in a 5% CO_2_ atmosphere.

Normal peritoneal mesothelial cells MES-F were purchased from Tebu-bio, pulmonary fibroblasts CCD-19Lu from the ATCC-LGC Standards, and pulmonary endothelial cells HMVEC-L from Lonza. These cells were cultured in their specific media according to the manufacturers' recommendations. The bronchial epithelial cells were obtained and cultured as previously described [35]. Cells were routinely checked for *Mycoplasma* contamination using the PlasmoTest^TM^ from InvivoGen.

### MV infection

Live-attenuated Schwarz vaccine strain of measles virus (MV), MV recombinant for the enhanced green fluorescent protein (MV-eGFP) and MV recombinant for the cherry protein (MV-ch) were produced and purified as previously described [36]. Infection of cells with the different measles virus vaccinal strains lasted 2 hours at 37°C. Viral inoculum was then replaced by fresh culture medium, unless otherwise indicated.

### MV replication assay

A day before infection, cells were seeded in 96-well plates, at a density of 5,000 cells/well for the MPM cell lines, and at the recommended density for each healthy cell type. Different multiplicities of infection (MOI) were used for infection with MV-ch (0.1, 1 and 10). Fluorescence at 610nm was analyzed every day during 10 days using a ChemiDoc™ MP imaging system (Bio-Rad). Quantification was performed using Image Lab 4.1 Software (Bio-Rad) with the relative fluorescence corresponding to the ratio between the fluorescence measured in infected cells and the non-infected cells. The medium was replaced every 3 days to match the conditions of the cell viability assay. For the CD46 blocking assay, we used a CD46-specific mAb (clone M177, Hycult Biotech) or a mouse IgG1 isotype as a control (clone MOPC-21, Biolegend) at a final concentration of 10μg/mL. These antibodies were added 30 minutes before adding the MV-ch at an MOI = 1. To test the inhibition of MV replication with type I IFN, we added rhIFN-alpha-2a and rhIFN-beta-1a (ImmunoTools) at concentrations ranging from 10IU/mL to 10,000IU/mL, 4 hours before the infection with MV-ch at MOI = 1, and the viral inoculum was not replaced for this assay.

### Cell viability assay

Cells were seeded in 96-well plates and infected with MV as described in the previous paragraph. Cell viability was measured using Uptiblue reagent (Interchim), an oxidation-reduction sensor that indicates cell metabolic activity. At days 3, 6 and 9 after the infection, the Uptiblue reagent was added (5%, v/v) into the culture medium for 2 hours at 37°C. Fluorescence was then measured at 590nm using a ChemiDoc™ MP imaging system (Bio-Rad). Quantification was performed using Image Lab 4.1 Software (Bio-Rad) and the viability was expressed as a percentage compared to non-infected cell viability. Culture medium containing Uptiblue was then replaced by fresh medium to continue the kinetic experiment.

### Video microscopy

A day before infection, cells were seeded in 24-well plates, at a density of 10^5^cells/well for the MPM cell lines, and at the recommended density for healthy cells. Cells were infected with MV-eGFP (MOI = 1). The time-lapse video microscopy was performed using a Leica DMI6000B microscope with a 10x objective. Images were acquired every 15 or 30 minutes for 3 to 4.5 days. We used MetaMorph^®^ Microscopy Automation & Image Analysis Software (version 7.8) and Fiji Software for acquisition and analysis.

### Flow cytometry

To measure CD46 expression on the cell surface, we stained the cells with a FITC-conjugated anti-CD46 mAb (clone E4.3, BD Biosciences). Fluorescence was measured on FACS Calibur (BD Biosciences) and analyzed using FlowJo software. The results are expressed as relative mean of fluorescence intensity (RMFI). To measure Mx1 in the cytoplasm 72 hours after infection with MV at MOI = 1, cells were fixed with PBS containing 4% paraformaldehyde for 10 minutes at room temperature. Cell washes and mAb dilutions were performed with PBS containing 0.1% BSA (bovine serum albumin) and 0.1% saponin. Unconjugated anti-Mx1 mAb (clone M143, Dr. Georg Kochs, University Medical Center Freiburg) and, as a control, an unconjugated mouse IgG2a isotype (clone MOPC-173, Biolegend) were used. A DyLight^TM^488-conjugated anti-mouse IgG antibody (clone Poly4053, Biolegend) was used as secondary antibody. The results are expressed as RMFI.

### Real-time RT-qPCR

MPM cell lines and healthy cells were seeded in 6-well plates at a density of 0.5×10^6^cells/well and infected with MV at MOI = 1. 72 hours after infection, total cell RNA was extracted using the Nucleospin^®^ RNA II kit (Macherey-Nagel) and 0.5μg total RNA was reverse transcribed using MMLV reverse transcriptase (Invitrogen). PCR reactions were conducted using QuantiTect primer assays (Qiagen) and Maxima SYBR Green/ROX qPCR Master Mix (Fisher Scientific) according to the manufacturer's instructions. Gene expression was analyzed in non-infected and infected cells using QuantiTect primers pairs for *IFNA1* (coding for IFN-α), *IFNB1* (IFN-β), *IFNL1* (IFN-λ1), *Mx1* (Mx1), *DDX58* (RIG-I) and *IFIH1* (MDA5). The gene expression was expressed as relative expression compared to the expression of a housekeeping gene that encodes human large ribosomal protein (RPLPO).

### Cytokine detection

IFN-α and IFN-β production were measured by ELISA (MabTech and PBL Assay Science, respectively), according to the manufacturer's instructions. Supernatants of MPM cell lines and healthy cells were collected 72 hours after infection with MV at MOI = 1 and used directly for ELISA without freezing.

### Statistical analysis

Statistical analysis was performed using GraphPad Prism 5 software (GraphPad Software Inc.). To compare two groups, a nonparametric, one-sided, unpaired Mann-Whitney comparison test was used. For statistical analysis comparing more than two groups, nonparametric one-way ANOVA (Kruskal-Wallis test) was used, with Dunn's post-test. All data are presented as mean±SEM. P values less than 0.05 were considered to be statistically significant. *p < 0.05, **p < 0.01, ***p < 0.001.

## SUPPLEMENTARY MATERIAL FIGURES AND VIDEOS
























