# Supplementary material for: Sensitivity of human pleural mesothelioma to oncolytic measles virus depends on defects of the type I interferon response
Source: Oncotarget. 2015 Nov 2;6(42):44892–904. doi: 10.18632/oncotarget.6285 (PMC4792599; doi:10.18632/oncotarget.6285)
Supplement: Supplementary file 1 [file oncotarget-06-44892-s001.pdf]

Sensitivity of human pleural mesothelioma to oncolytic measles virus depends on defects of the type I interferon response

Supplementary Material

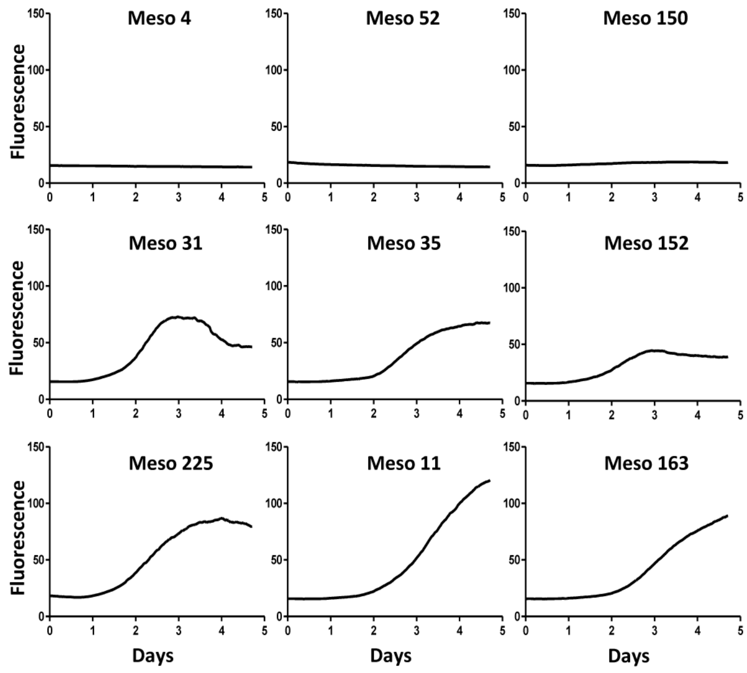

Supplemental figure 1. MV replication measured by time-lapse video microscopy. MPM cell lines were infected with MV-eGFP at MOI=1. Fluorescence microscopy video was performed with a picture taken every 30 minutes with a Leica DMI6000B microscope with a 10x objective. The fluorescence values correspond to the mean of GFP fluorescence measured per field. Results are expressed as the mean of four randomly selected fields.

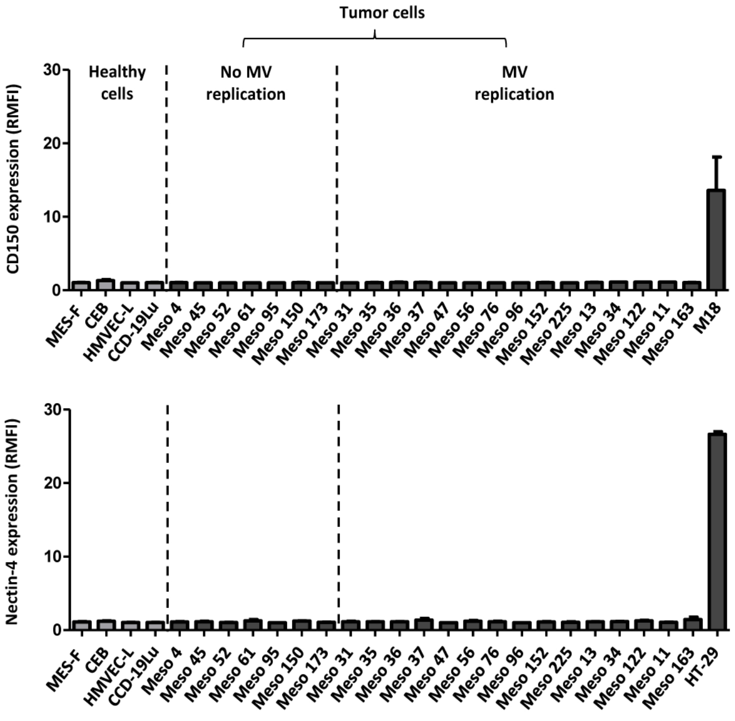

Supplemental figure 2. MPM tumor cell lines and healthy cells do not express CD150 and nectin-4 at their surface. Expression of CD150 and nectin-4 was measured on the cell surface by flow cytometry. Melanoma M18 and colorectal adenocarcinoma HT29 tumor cells were used as positive controls for the expression of CD150 and nectin-4, respectively. The results are expressed as the RMFI mean±SEM of three independent experiments.

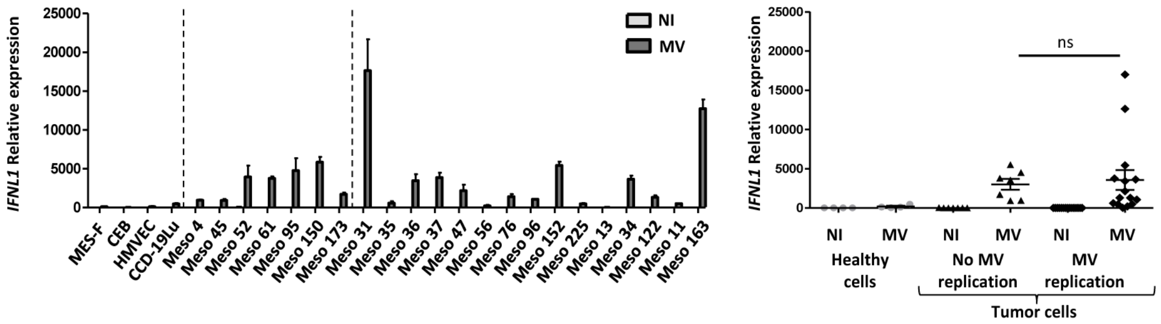

Supplemental figure 3. The sensitivity to MV infection does not depend on defects of the antiviral type III IFN response. The expression of the gene *IFNL1* coding for IFN-λ1, implicated in the antiviral type III IFN response, was analyzed by RT-qPCR 72h after MV infection of tumor and healthy cells (MOI=1). The expression is expressed as relative expression compared to *RPLPO* gene expression. Non-infected cells (NI) are in light gray and infected cells (MV) are in dark gray. A histogram shows the expression by each cell line, and a scatter plot shows the expression by groups (healthy cells, tumor cells with no MV replication, tumor cells with MV replication). Results are expressed as the mean ±SEM of three independent experiments. * $p<0.05$; ** $p<0.01$; *** $p<0.001$, one-way ANOVA (Kruskal-Wallis).
